# Circulating Tumor Cells for the Management of Renal Cell Carcinoma

**DOI:** 10.3390/diagnostics8030063

**Published:** 2018-09-03

**Authors:** Lucile Broncy, Patrizia Paterlini-Bréchot

**Affiliations:** 1 INSERM Unit 1151, Faculté de Médecine, Université Paris Descartes, 75014 Paris, France; lucile.broncy@rarecells.com; 2 Laboratoire de Biochimie A, Hôpital Necker-Enfants Malades, 75015 Paris, France

**Keywords:** circulating tumor cells (CTC), clear cell renal cell carcinoma (ccRCC), liquid biopsy, circulating cancer cells (CCC), Isolation by Size of Tumor cells (ISET)

## Abstract

Renal cell carcinoma is a highly malignant cancer that would benefit from non-invasive innovative markers providing early diagnosis and recurrence detection. Circulating tumor cells are a particularly promising marker of tumor invasion that could be used to improve the management of patients with RCC. However, the extensive genetic and immunophenotypic heterogeneity of cells from RCC and their trend to transition to the mesenchymal phenotype when they circulate in blood constitute a challenge for their sensitive and specific detection. This review analyzes published studies targeting CTC in patients with RCC, in the context of the biological, pathological, and molecular complexity of this particular cancer. Although further analytical and clinical studies are needed to pinpoint the most suitable approach for highly sensitive CTC detection in RCC patients, it is clear that this field can bring a relevant guide to clinicians and help to RCC patients. Furthermore, as described, a particular subtype of RCC—the ccRCC—can be used as a model to study the relationship between cytomorphological and genetic cellular markers of malignancy, an important issue for the study of CTC from any type of solid cancer.

## 1. Introduction

Renal cell carcinoma (RCC) is a very invasive and chemoresistant disease which is often treated by surgical resection as it also responds poorly to radiotherapy [1]. Importantly, more than 30% of localized RCC recur or metastasize after treatment [2]. Even in RCC cases believed to be curable by radical nephrectomy, distant metastasis can develop 5–10 years after surgery [3]. Non-specific immunotherapies using cytokines have been widely employed in past decades to treat metastatic RCC but, due to their limited success in improving median survival of patients, they are now being gradually replaced by targeted immunotherapies [4]. Currently, available targeted therapies for metastatic RCC, such as immune checkpoint inhibitors, mTOR inhibitors, or VEGF tyrosine kinase inhibitors, are routinely administered in clinical practice, yet no predictive biomarkers are used to guide the selection of those targeted treatments [5]. In this context, there is an urgent need for reliable biomarkers of RCC, enabling early diagnosis, prognosis, and monitoring of treatment efficacy and potential relapse of the disease.

Liquid biopsies offer a promising perspective for non-invasive and repeatable assessment of the tumor burden [6]. From protein profiling in urinary exosomes [7] to non-coding circulating RNA screening in plasma or serum of RCC patients [8], liquid biopsies encompass a broad range of cytological and molecular analyses performed on biological fluids. In particular, studying circulating tumor cells (CTC) and cell-free tumor DNA (ctDNA) has revealed tremendous potential to improve cancer patients’ care worldwide [9]. Notably, ctDNA has shown potential value as a predictive biomarker of response to immune checkpoint inhibitors for metastatic RCC patients [10]. Analysis of ctDNA presents as a straightforward approach for genetic assessment of the tumor burden (for a comprehensive review of the role of ctDNA in the management of RCC, please refer to [11]). However, when it comes to localized RCC tumors, ctDNA assessment has been reported as particularly difficult [12] as compared to other types of solid tumors [13]. Moreover, CTC are uniquely suited to interrogate functional heterogeneity by combining genetic and transcriptomic assessment of single CTC [14] or by parallel single-cell transcriptome and epigenome analysis [15]. Yet, few studies have reported on CTC analysis in the context of RCC.

## 2. Materials and Methods

The present review, which is not meant to be exhaustive, was prepared by gathering studies focused on the analysis of CTC in the context of RCC. To that aim, we performed PubMed searches using the following keywords: “liquid biopsy” & “renal cell carcinoma” & ”kidney cancer”, or “circulating tumor cells” & “renal cell carcinoma” & “kidney cancer”. Studies and reviews on liquid biopsy that did not concern RCC, as well as RCC studies that did not report on CTC were excluded from the systematic review, although some are cited as reference for particular arguments within the text. A total of 12 publications were selected and included to the systematic review on CTC studies, as shown in Table 1. Additionally, an overview of the pathological and molecular features of RCC is proposed, as basis for the molecular strategies described in the reviewed CTC studies.

## 3. Renal Cell Carcinomas (RCC)

Renal cell carcinomas (RCC) are the most frequent types of kidney cancer, accounting for 5% of new cases of cancer in men and 3% in women for the year 2017 in the US [27]. RCC do not constitute a single cancer entity but rather encompass a broad range of phenotypically and genetically distinct kidney neoplasms among which the three most common subtypes are clear cell (ccRCC) accounting for an estimated 75% of cases, papillary (pRCC) accounting for approximately 10%, and chromophobe carcinomas (chRCC) found in roughly 5% of cases [28]. The more frequent RCC subtype is referred to as “clear cell” carcinoma because of high lipid content in the cytoplasm of ccRCC cells which is washed away during staining procedures, resulting in observation of unstained or faintly eosinophilic cytoplasms during pathological examination [29]. Clear cell RCC tumors typically present with a highly vascularized stroma and frequently show hemorrhagic areas [30]. Early stage ccRCC is mostly discovered by accident during unrelated examinations since the majority of cases remain asymptomatic before metastatic progression [31]. Several other subtypes exist, such as the rarer collecting duct RCC, and the continually evolving classification of renal tumors from the world health organization (WHO) was recently reformed by the 2016 update to add five new entities to the 10 previously established subtypes [32]. Despite increasingly precise and complex subtype classification guidelines, approximately 4% of RCC remain unclassified and those uRCC tumors are currently emerging as particularly aggressive types of renal malignancies [33]. The biological behavior of renal tumors remains largely unpredictable by histology alone.

**Figure 1:**
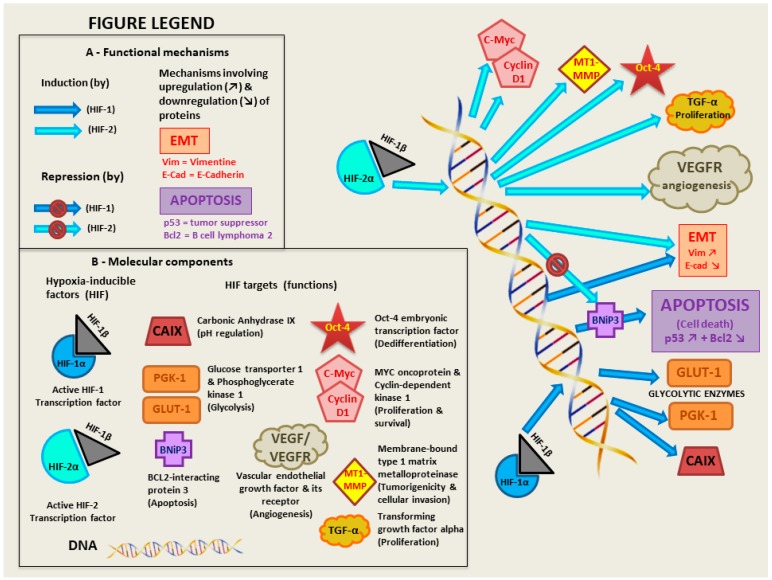
Molecular targets of hypoxia-inducible factors (HIF) in human kidney cells during hypoxia or in absence of functional VHL protein.

Deletion on one allele of the short arm of chromosome three harboring the von Hippel–Lindau (VHL) gene locus (3p25) has been described to occur at exceptionally high rates in large ccRCC cohorts, characterizing up to 94% of cases [34]. Loss of VHL function in humans has been correlated to cellular dedifferentiation, illustrated by a gain of expression of the mesenchymal marker vimentin in epithelial kidney cells [35]. This process is characteristic of an early epithelial-to-mesenchymal transition (EMT) which favors acquisition of invasive properties of kidney cells and is induced by the biallelic inactivation of the VHL gene in humans [36]. Indeed, the VHL protein (pVHL) was first characterized as an effector mediating the proteolytic degradation of alpha subunits of hypoxia-inducible factors (HIF-1αand HIF-2α) through binding of elongins B and C as stabilizing cofactors [37,38]. On its own, this hypoxia-related function of pVHL has tremendous biological impacts since HIF are potent transcription factors known to target over 800 genes [39]. It is interesting to note that some missense VHL mutations may favor HIF-2 accumulation while others result in activation of both HIF-1 and HIF-2 since those transcription factors target different genes [40]. A brief description of the main HIF targets is presented in Figure 1.

Aside from the biallelic inactivation of VHL, according to recent studies, common genetic alterations that are most recurrently found in ccRCC patients often target genes controlling the expression of epigenetic regulator proteins involved in modulating chromatin conformation and accessibility to transcription, such as PBRM1, SETD2, BAP1, ARID1A, KDM5C, and KDM6A [41]. Interestingly, studying human biology has revealed that PBRM1, BAP1, and SETD2 are all located in proximity of the VHL locus on the short arm of chromosome 3 and represent simultaneous targets of depletion by LOH at 3p [34]. In a cohort of 227 ccRCC patients, truncating mutations of PBRM1 impacting the chromatin remodeling function of the encoded protein were found in 41% of cases [42]. Furthermore, concomitant inactivation of VHL and PBRM1 was recently reported to display synergistic effects of metabolic deregulation in ccRCC cell lines in vitro [41]. Additionally, expression of functional PBRM1 has been shown to restrain VHL loss-driven ccRCC progression, thus illustrating the existence of distinct subtypes of VHL-null ccRCC [43].

**Figure 2:**
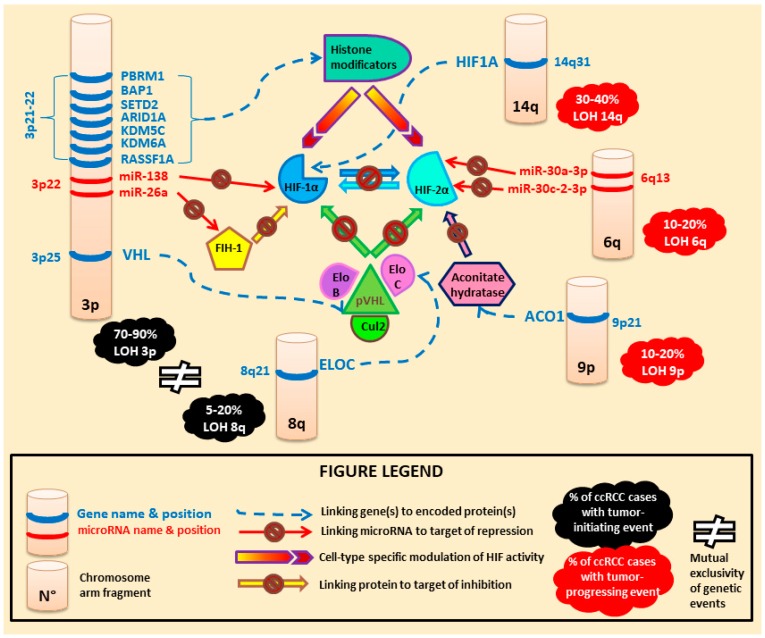
Genetic and epigenetic regulation of hypoxia-inducible factors (HIF) in human kidney cells.

Conversely, in tumors displaying functional pVHL expression, high throughput molecular studies have shown that activation of the oncogenic pathway related to HIF-α transcription factors can be achieved through distinct genetic alterations that display mutual exclusiveness with inactivating VHL mutations. For instance, mutations in the ELOC gene (also called TCEB1) together with LOH at its 8p locus were found to inactivate the elongin C cofactor of pVHL in 40% of ccRCC patients with wild type pVHL expression [34]. An overview of the oncogenic pathway related to HIF activation is presented in Figure 2.

Intratumoral heterogeneity (ITH) is a prominent feature of ccRCC, as evidenced by several groups using multiregional sequencing of ccRCC tumorous and metastatic tissues compared to sequencing of adjacent normal kidney tissue [44,45,46]. For instance, using multiregion sampling and high throughput deep sequencing, Gerlinger et al. determined that 75% of all driver aberrations found in each ccRCC patient were subclonal while VHL alterations, including LOH at 3p and epigenetic silencing, were the only ubiquitous events found across all 79 tumor sites sampled from 10 ccRCC patients [46]. Such observations are consistent with the role of VHL inactivation as a critical founder event in the majority of ccRCC cases.

Nonetheless, for the minority of ccRCC cases that do not present with LOH at 3p or other genetic and/or epigenetic aberrations affecting VHL transcription, understanding the subclonal organization of genetic, epigenetic, and transcriptomic features that sustain tumor growth and invasion seems crucial to improving medical care for patients [47]. It is now well accepted that single-cell studies are best suited to uncover the extent of ITH in a given patient. However, very few publications have reported single-cell analyses in the context of RCC. For instance, Xu and colleagues were the first to publish single-cell exome sequencing results in the context of ccRCC [48]. By comparing single-cell data obtained from one patient, the authors determined that, although this particular ccRCC tumor did not bear genetic alterations of either VHL or PBRM1, over 70% of the alterations found were cell-specific while less than 30% were common to multiple cells within the tissue [48].

In the meantime, using single-cell RNA sequencing (RNA-seq), Kim et al. examined the intratumoral heterogeneity of a pair of primary RCC tumor and its lung metastasis [49]. This study demonstrated a considerable variability, in terms of activated drug target pathways, between a given primary tumor and its corresponding metastasis, as well as among individual cancer cells within each sample site. By combining two therapeutic strategies to simultaneously target two mutually exclusive pathways, the authors demonstrated a significant increase in treatment effectiveness compared to monotherapy using patient-derived xenograft platforms in vitro and in vivo [49]. Such studies highlight the potential of RNA profiling at the single-cell level to provide a comprehensive picture of functional heterogeneity and identify specific signatures of drug response within subpopulations of cancer cells, thereby improving medical care for patients.

More recently, Li and colleagues performed single-cell exome sequencing in a cohort of 57 RCC patients to uncover the molecular characteristics of renal cancer stem cells [50]. Results from this study allowed to demonstrate that RCC cells expressing CD133 possess stem cell properties and likely originate from renal cancer cells instead of normal renal cells. Additionally, these authors identified novel renal cancer stem cell driver mutations, affecting pathways of DNA damage repair and RNA-binding proteins, which could serve as important prognostic factors and therapeutic targets for RCC in the future [50].

It is noteworthy that ccRCC is an aggressive form of RCC [32]. Evidence of long dormancy and high metastatic potential of ccRCC, together with frequent intravenous tumor embolization sometimes extending to the inferior vena cava [51], suggest that circulating tumor cells (CTC) may represent interesting prognostic and predictive markers to monitor disease progression as well as patients’ response to therapy.

## 4. CTC Collection/Detection in Renal Cell Carcinoma

Only few studies have been published on CTC analysis in patients with sporadic ccRCC (see Table 1).

It has to be pointed out that CTC belong to the larger group of circulating rare cells (CRC), which are heterogeneous non-hematological cells found in small proportions in blood. They include circulating non-tumor epithelial cells, circulating tumor (mesenchymal, epithelial, and epithelial/mesenchymal) cells and circulating endothelial cells [52]. The “reference” cytopathological criteria, extensively validated by Hofman et al. [53,54], and complemented by immunolabeling, allow to distinguish the different types of CRC. The findings that non-tumorous epithelial cells are present in a proportion of cancer patients [53,54] and that non-tumorous circulating epithelial cells are also found in patients with inflammation and benign diseases of a non-neoplastic nature [55,56,57,58] warrant caution when employing the term circulating tumor cells (CTC) to designate CRC extracted from blood by epithelial marker-dependent methods without any other cellular diagnostic approach. Consequently, we have introduced the term circulating cancer cells (CCC) to strictly designate CRC isolated from blood without bias and diagnosed by cytopathology as tumor cells [52].

Given the complexity of the CRC field, it is not surprising that discrepancies can be found when comparing results derived from different studies, using different methods of CTC collection and identification. We therefore chose to group the reviewed studies depending on the methods used for CTC enrichment and analysis. However, a detailed overview of all the methods available for CTC collection/detection remains out of the scope of the present review (for a comprehensive review of CTC isolation and detection methods, please refer to [59]).

### 4.1. Epithelial Marker-Dependent Isolation/Detection of CTC in Renal Cell Carcinoma

Importantly, cancer cells from ccRCC patients are prone to EMT [35,36] and often lack epithelial antigens, which may impair their capture from blood and analysis when epithelial marker-dependent collection/detection methods are used. For example, using the CellSearch system (relying on the epithelial marker known as EpCAM for selection), Gradilone et al. detected CTC only in 16% of 25 metastatic ccRCC patients [21]. Additionally, Allard and colleagues applied CellSearch to 11 metastatic RCC patients and found very few CTC (mean number of 1 CTC for all patients) [17]. Among 145 healthy women tested in the latter study, 8 of them (5.5%) showed 1 CTC detected by CellSearch while 14 of 199 (7.5%) women with benign or non-malignant diseases had 1 CTC detected by CellSearch. These results defined a cutoff of ≥2 CTC to be used to count CTC. Importantly however, this cutoff was not reached for 75% of RCC patients tested, demonstrating the inadequacy of CellSearch to provide reliable CTC counts in RCC patients [17]. Consistently, using immunostaining of cytokeratins (CK8/18) after hematopoietic cell depletion by AutoMacs, Bluemke et al. found positive expression of CK8/18 in only 4.5% of 154 RCC patients while blood samples from another 38% of patients harbored CK-negative cells with large blue-stained nuclei [20].

Surprisingly, a recent case report describing the use of an EpCAM-based microfluidic isolation of circulating epithelial cells by CytoQuest CR (Abnova, Taipei, Taiwan) and cytokeratin positivity, as a marker for CTC detection, managed to correlate CTC kinetics with response to sunitinib treatment of a metastatic RCC patient over a period of 20 days [25]. Although, said correlation emerged solely from the observation that, while CTC numbers dropped below baseline count at Day 20, CTscan examination on Day 128 showed a 14% reduction in the size of the primary renal lesion with concomitant shrinkage of the lung metastasis. This study also evidenced a surge of CTC numbers at Days 2 and 10 following the start of sunitinib treatment and possibly related to potential oncolytic events. However, the patient experienced Grade 3 asymptomatic hypertension according to the Criteria for Adverse Events (NCICTCAE, version 4.0) on Days 2 and 10 while sunitinib administration remained constant in term of dosage. An antihypertensive drug (amlodin 5 mg) was prescribed on Day 2, and the patient’s blood pressure returned to normal after Day 10. Therefore, CTC kinetics presented by Nagaya and colleagues appear best correlated to blood pressure variations rather than oncolytic effects. Indeed, circulating epithelial cell numbers have been shown to correlate with inflammation status in benign colon diseases [54] while circulating endothelial cells, which can also express cytokeratins, are known to correlate with pulmonary hypertension [60]. Therefore, the study by Nagaya et al. raises further issues concerning the specificity of cytokeratin-based CTC counting, especially since their case report did not include any healthy controls [25].

### 4.2. Other Marker-Dependent Isolation of CTC in Renal Cell Carcinoma

More recently, Liu and colleagues used the NanoVelcro platform combined with CA9-/CD147-capture antibodies to study CTC from 70 ccRCC patients [24]. This study demonstrated a significant association of CTC numbers as well as the CTC expression status of Vimentin, a mesenchymal marker that is also present on a subset of endothelial cells, with disease progression, including pathologic features and clinical staging. Indeed, these authors performed a comprehensive analysis of CA9 and CD147 expression patterns in resected ccRCC tumors from their patient cohort, evidencing CA9 and/or CD147 expression in 97.1% of patients while EpCAM was only detected in 18.6% of ccRCC tumors. However, it is important to note that CA9 mRNA was found to be expressed in normal liver and biliary tissues [16] while CD147, a member of the immunoglobulin superfamily also called Basigin (BSG), is ubiquitously expressed in 15 distinct normal tissues [61] and can serve as a marker of renal fibrosis or IgA nephropathy [62]. Altogether, these observations drawn from the literature point to a poor specificity of both CA9 and CD147 to select for renal cancer cells in the bloodstream.

### 4.3. RT-PCR-Based Methods for CTC Interrogation in Renal Cell Carcinoma

Earlier studies have also looked for CTC in the blood of RCC patients using various RT-PCR approaches. McKiernan and colleagues, for instance, detected CA9 gene expression (coding for the Carbonic Anhydrase IX protein, or CAIX) in mononuclear cells extracted from blood by density gradient from 49% of 37 RCC patients and from 1.8% of 54 healthy controls [16]. Those results illustrate the lack of specificity of CA9 gene expression to identify CTC in RCC blood samples, as CAIX can also be expressed in hypoxic or necrotic tissues regardless of their tumor origin. In fact, CAIX is a specific marker of HIF-1 accumulation as the CA9 gene encoding CAIX is transcriptionally activated by the HIF-1 transcription factor [63,64]. Indeed, HIF-1 accumulation characterizes a metabolic switch induced by the complete loss of functional pVHL. However, VHL alterations favoring HIF-2 accumulation would not cause this CAIX-positive phenotype among ccRCC patients [65]. Furthermore, other RCC subtypes are not associated with LOH at 3p nor with CAIX expression [32].

Another approach based on molecular data consists in detecting VHL gene alterations present in the peripheral blood of ccRCC patients. This analysis was done by Ashida et al. in a study where mutation-specific primers tailored to each individual ccRCC patient were designed for nested RT-PCR targeted to the pooled mononuclear cells extracted from blood by density gradient centrifugation. The authors reported a 75% concordance of the VHL gene alterations detected in peripheral blood with those obtained on matched tumor samples [3]. However, this approach is time-consuming as it requires the synthesis of specific molecular probes tailored to the mutation(s) found in the tumor tissue of each individual patient, and it would be difficult to implement in a routine clinical setting. Furthermore, as other RT-PCR assays, it cannot provide the CTC count.

Later on, Cadherin-6 gene expression was used as a marker of CTC presence in ccRCC blood samples. Li and colleagues identified Cadherin-6 gene expression by RT-PCR in 45.7% of 46 ccRCC patients and in none of 25 healthy donors [18]. Despite the absence of Cadherin-6 expression in healthy donors from the latter study, the specificity of such an RT-PCR assay remains insufficient since Cadherin-6 expression characterizes circulating epithelial cells without further indication regarding their tumorous nature [66]. In contrast, Burczynski et al. opted for a more global RT-PCR approach, searching for multivariate gene expression patterns in mononuclear cells extracted from blood by density gradient centrifugation to correlate with disease progression and survival of 45 RCC patients treated by mTOR kinase inhibitor [19]. These authors, after analyzing hybridized oligonucleotide arrays of over 12,600 human sequences, reported a panel of 20 genes enabling overall performance accuracies of 72% for predicting time of death and 85% for predicting progressive disease. Yet, because of the absence of a placebo or active control arm in their clinical trial, the authors were unable to determine whether those gene expression patterns were predictive of clinical outcome only in the context of the specific mTOR kinase inhibitor evaluated as therapy [19].

### 4.4. Negative CTC Enrichment by Hematopoietic Cell Depletion in Renal Cell Carcinoma

On the other hand, several studies using density gradient blood centrifugation to recover mononuclear cells, followed by hematopoietic cell-depletion, have reported results on CTC identification when in fact those results profiled circulating rare cells (CRC) of heterogeneous nature, possibly including circulating stem cells and non-tumor cells of epithelial and/or endothelial origin [20,23]. The recent report by Nel et al. uncovered an extensive phenotypic heterogeneity among CRC derived from individual patients [23]. Interestingly, using both phenotyping- and gene expression-focused molecular approaches, these authors were able to correlate the presence of mesenchymal (*N*-cadherin-positive) and stem-like (CD133-positive) cells in blood with a decreased expression of genes coding for the first alpha subunit identified within hypoxia-inducible factors (HIF-1α) and the receptor for the vascular-endothelial growth factor (VEGFR) as well as with shorter progression-free survival (PFS) among the 14 metastatic RCC patients tested, including 12 ccRCC. However, the lack of proper distinction between CTC and CRC, exemplified by the finding of ‘false’ CTC in healthy controls, prompted Nel et al. to apply statistical cut-offs in order to help counting CTC in 64% of metastatic RCC patients exhibiting panCK-positive CRC [23].

Furthermore, using four distinct ccRCC cell lines spiked in healthy donor’s blood, Maertens and colleagues demonstrated the superior recovery rates obtained by cell size-based enrichment (Parsortix, Angle PLC) over EpCAM-based collection of tumor cells and CD45-mediated depletion of leukocytes [67].

### 4.5. Cell Size-Based Enrichment, Morphological and Genetic Detection of CTC in Renal Cell Carcinoma

El-Heliebi et al. have isolated CRC from RCC patients by blood filtration (size-based) and used a combination of morphological criteria and genetic analysis in the aim of identifying malignant cells [22]. Their conclusion is that, for patients with renal tumors, cytomorphological classification alone is not sufficient to allow for reliable detection of single CTC or clusters of CTC, also called circulating tumor microemboli (CTM), and needs immunocytochemical and/or molecular methods. However, the authors do not provide any guide for identifying CTC or CTM as, in their study, molecular results are not consistent with immunological nor immunomorphological data. They found that all the CRC clusters identified in 30 RCC patients, including 25 ccRCC and 5 papillary RCC, were of endothelial origin, based on their CD31 positivity and only one was of tumor origin, based on carbonic anhydrase nine (CAIX) positivity, used as a marker of RCC-derived tumor phenotype. Furthermore, out of 12 CRC-clusters studied by GCH array, including 6 with tumor phenotype, only 2 showed CGH abnormalities, including one with tumor phenotype and one with uncertain phenotype, but in both cases the genomic abnormalities were different from those detected in the tumorous tissue. The authors thus failed to provide any guideline concerning detection of circulating tumor cells and tumor microemboli in patients with RCC [22].

Induction of angiogenesis is an important step towards cancer invasion. The so called “angiogenic switch” is considered to be a major stepping stone in the transition from primary non-invasive neoplasms to invasive carcinomas [68]. Hypoxia signaling within the tumor triggers vascular endothelial growth factor (VEGF) secretion by tumor cells and elevated levels of active VEGF induce disorganized vessel sprouting and reduced pericyte coverage, leading to a sustained immature vascular network [69]. In the context of ccRCC, the observed metabolic deregulation affecting hypoxia signaling could in part explain the dense vascularization observed in these tumors [70]. Interestingly, Yang and colleagues analyzed RCC and hemangioblastoma tumors associated with loss of pVHL function and discovered an inverse correlation between pVHL levels and CD31 expression, suggesting that CD31 expression may not be restricted to non-malignant endothelia and could also occur in tumor cells [71]. Consequently, RCC-derived tumor cells with low expression of pVHL should be further investigated regarding possible concomitant expression of CD31.

Furthermore, Kats-Ugurlu et al. analyzed tumor fragments in renal venous outflow of 42 ccRCC patients and determined that 33% of them harbored circulating clusters containing a core of cancer cells surrounded by an external coating of endothelial cells [72]. Importantly, this report confirms previous observations made by Sugino et al. regarding CD31-positive endothelia-coated tumor microemboli observed inside large blood vessels on sections of RCC tissues [73].

In fact, the non-consistent CGH array results obtained on circulating cell clusters by El-Heliebi and colleagues are puzzling and difficult to explain [22]. They could in part reflect the analysis of tumor-derived circulating endothelial cell clusters, since those circulating clusters have been described by Cima and colleagues to bear cytomorphological anomalies without corresponding tumor-associated genetic alterations in the context of early stage colorectal cancer [74]. Additionally, those results could be related to the mixtures of endothelial and cancer cells, also possibly including other non-tumor cells in the cluster like pericytes, smooth muscle cells, and immune and stromal cells, as described by Kats-Ugurlu et al. in ccRCC blood samples [72,75], in agreement with other publications focused on resected human ccRCC tumors [73] or murine xenograft models [76]. As suggested in the literature [77,78,79], different quantitative contributions of each cell type to those heterogeneous clusters could potentially give rise to both balanced and unbalanced CGH array results such as those found by El-Heliebi and colleagues. Taken together, these arguments challenge the conclusions of El-Heliebi and colleagues [22] and stimulate to perform further investigations.

We recently used ISET filtration and cytopathological analysis to study CRC in 30 ccRCC patients [26]. Among the 205 CRC identified by cytopathology in the blood of 29 patients, 141 cells from 29 patients were classified as CRC with uncertain features of malignancy (UMF) while 64 cells derived from 20 patients were diagnosed as CCC.

We aimed at comparing the cytopathological analysis of CRC with their single-cell genetic analysis targeted to VHL mutations in order to assess the specificity and sensitivity of cytopathology [26]. The presence of a VHL mutation was validated by blind molecular analysis in 57 of the 64 CCC and in 125 of the 141 CRC-UMF. The remaining 7 CCC and 16 CRC-UMF without VHL mutation were all found to derive from the 4 patients harboring a wild type VHL sequence in their tumor tissues.

Our molecular results thereby showed a complete correlation of absence of VHL mutation in the tumor and in the corresponding CCC and CRC-UMF derived from the same 4 patients. However, in the absence of VHL mutation, those 23 CRC (7 CCC and 16 CRC-UMF) could not be used to assess the sensitivity of the cytological diagnosis. Remarkably, all the remaining 57 CCC as well as 104 CRC-UMF exhibited identical VHL mutations as those detected in the corresponding tumor samples, indicating the neoplastic nature of cells classified as CRC-UMF by the cytopathologists. Additionally, 21 CRC-UMF derived from 11 patients were found to display a distinct VHL mutational profile than that found in the corresponding tumor tissue, raising questions regarding their possible tumor or pre-tumor nature. It is striking that, all the CCC identified by “reference” cytopathological criteria [75,76] in our blind analysis of CRC from ccRCC patients were carrying identical VHL gene sequences as those found in the corresponding tumorous tissues, while no VHL mutation was found in all the leukocytes tested as controls. Such results are consistent with a complete (100%) specificity of the cytopathological approach. Additionally, 104 CRC classified as CRC-UMF by the cytopathologists were also found to carry identical VHL mutations as those detected in the corresponding tumorous tissues.

We consider that, since we found VHL mutations identical to those found in the tumor tissue in CRC having uncertain malignant features (CRC-UMF) and having acquired the capability to circulate in blood, those CRC with VHL mutation are in fact tumor cells. Thus, among the 161 single cells found with the same VHL mutation detected in the tumor tissue, 57 (35%) have been identified as CCC by cytopathological analysis and 104 (72%) have been identified as cancer cells by genetic analysis. As a consequence, cytopathology has 35% sensitivity in our study. If we consider patients with CCC identified by cytopathology, the sensitivity is 72%. This result suggests that genetically-based identification of tumor cells could be more sensitive than their cytopathological detection, independently from the tumor type from which they originate.

We also found 21 CRC-UMF derived from 11 patients and exhibiting a distinct VHL mutational profile than that found in the corresponding tumor tissue, raising questions regarding their possible tumor or pre-tumor nature. It will be important to broaden the scope of future molecular characterization of CRC in ccRCC by analyzing additional molecular markers implicated in ccRCC tumorigenesis in order to achieve diagnostic significance of said molecular results.

To our knowledge, this study is the first to combine highly experienced cytopathological analysis according to the “reference” criteria [52,53] with blind single-cell genetic analysis of cells previously assessed by cytopathological study. This approach needed to overcome specific technical challenges. In fact, the DNA of fixed single cells is particularly difficult to analyze and cell-staining adds further technical difficulties, as illustrated by the 62.7% successful amplification of the three VHL exons from all the individual CRC that were microdissected in our study. 

With the continuous improvement of molecular testing applied to single-cell analyses, we can hope that in the future whole genome, transcriptome, and maybe proteome analyses of fixed and stained single cells could become available, thereby increasing our capacity to investigate CRC and detect their tumorous nature. Comparative studies targeting CRC isolated from patients with ccRCC and from patients with benign kidney diseases, including patients affected by hereditary VHL disease at the pre-cancerous stage, could also help to assess the diagnostic value of specifically targeted DNA mutations, including VHL mutations.

## 5. Conclusions

Renal cell carcinoma (RCC) is a highly invasive cancer which could benefit from reliable non-invasive biomarkers for early diagnosis and disease monitoring. Liquid biopsy, in particular the study of cell-free tumor DNA (ctDNA) and of circulating cancer cells (CCC), offer a promising tool for assessment of the tumor burden and tumor invasion capability. Other markers, such as protein profiling in urinary exosomes and non-coding circulating RNA are under investigation. Although further studies are needed to better explore the heterogeneity of circulating rare cells in patients with RCC and to increase the sensitivity of CTC and ctDNA detection, these efforts are expected to generate valuable markers for early diagnosis and better follow up of patients, allowing early and timely personalized treatment. Finally, the most frequent subtype of RCC—the ccRCC, which frequently carries the VHL mutation—can be used as a model to study, in the CCC and at the single cell level, the correlation between the cytomorphological and the genetic features of malignancy.

## Figures and Tables

**Table 1:** Selected studies on circulating tumor cells in renal cell carcinoma patients.

Reviewed Studies	CTC Collection Method	CTC Detection Method	Patients
McKiernan et al. 1999 [16]	Density gradient centrifugation	CA9 RT-PCR	9 metastatic RCC, 28 localized RCC, 5 benign renal lesions and 54 healthy controls
Ashida et al. 2000 [3]	Density gradient centrifugation	VHL mutation-specific PCR	29 sporadic ccRCC
Allard et al. 2004 [17]	CellSearch^®^ (EpCAM-based)	Cytokeratin expression	11 metastatic RCC, 199 benign diseases and 145 healthy controls
Li et al. 2005 [18]	Density gradient centrifugation	Cadherin-6 RT-PCR	11 metastatic RCC, 35 localized RCC and 25 healthy controls
Burzynski et al. 2005 [19]	Density gradient centrifugation	Global RT-PCR	45 advanced RCC
Bluemke et al. 2009 [20]	Density gradient centrifugation and immunomagnetic depletion of leucocytes by AutoMacs	Morphological assessment and cytokeratin expression by immunocytochemistry	154 RCC
Gradilone et al. 2011 [21]	CellSearch^®^ (EpCAM-based)	Cytokeratin expression	25 metastatic RCC
El-Heliebi et al. 2013 [22]	ScreenCell^®^ Cyto filtration	Morphological assessment	30 advanced RCC and 10 benign renal tumors
Nel et al. 2016 [23]	Density gradient centrifugation and hematopoietic cell depletion	Cytokeratin, *N*-Cadherin or CD133 expression by immunofluorescence	14 metastatic RCC and 14 healthy donors
Liu et al. 2016 [24]	NanoVelcro microfluidic platform (CA9-/CD147-capture antibodies)	Cellular diameter of 13–50 μm and positive cytokeratin expression	76 RCC, 10 benign renal tumors and 15 healthy controls
Nagaya et al. 2018 [25]	CytoQuest™ (EpCAM-based)	Cytokeratin expression without CD45 expression	1 RCC (case report)
Broncy et al. 2018 [26]	ISET^®^ filtration	Morphological assessment and single-cell VHL-targeted PCR	2 metastatic RCC and 28 localized RCC
